# Supplementary material for: MetaRibo-Seq measures translation in microbiomes
Source: Nat Commun. 2020 Jun 29;11:3268. doi: 10.1038/s41467-020-17081-z (PMC7324362; doi:10.1038/s41467-020-17081-z)
Supplement: Supplementary file 10 — Supplementary Data 7 [file 41467_2020_17081_MOESM10_ESM.zip › File2/Confidence_High_Taxonomy/159682_out.krona.html]

Javascript must be enabled to view this page.

members
magnitude
magnitudeUnassigned
count
unassigned
taxon
rank

159682\_out

15

2
superkingdom
15

phylum
15
1239

class
15
186801

186802
15
order

186803
family
15

genus
15
841

15
species
166486

SRS015694\_contig\_number\_6689SRS015854\_contig\_number\_1873SRS015960\_contig\_number\_contig-100\_50.126787SRS016335\_contig\_number\_16409SRS022137\_contig\_number\_22076SRS049959\_contig\_number\_contig-100\_5140.93657SRS056273\_contig\_number\_2794SRS063518\_contig\_number\_27687SRS076756\_contig\_number\_22358SRS103987\_contig\_number\_23359SRS140513\_contig\_number\_contig-100\_3563.42128SRS142923\_contig\_number\_contig-100\_1612.1613SRS148721\_contig\_number\_40613SRS148817\_contig\_number\_contig-100\_20756.60454SRS893252\_contig\_number\_15179
